# The First 250 ms of Auditory Processing: No Evidence of Early Processing Negativity in the Go/NoGo Task

**DOI:** 10.1038/s41598-020-61060-9

**Published:** 2020-03-04

**Authors:** Jack S. Fogarty, Robert J. Barry, Genevieve Z. Steiner

**Affiliations:** 1 Brain & Behaviour Research Institute and School of Psychology, University of Wollongong, Wollongong, NSW 2522 Australia; 2 NICM Health Research Institute and Translational Health Research Institute (THRI), Western Sydney University, Penrith, NSW 2751 Australia

**Keywords:** Cortex, Cognitive control, Perception, Neurophysiology, Human behaviour

## Abstract

Past evidence of an early Processing Negativity in auditory Go/NoGo event-related potential (ERP) data suggests that young adults proactively process sensory information in two-choice tasks. This study aimed to clarify the occurrence of Go/NoGo Processing Negativity and investigate the ERP component series related to the first 250 ms of auditory processing in two Go/NoGo tasks differing in target probability. ERP data related to each task were acquired from 60 healthy young adults (*M* = 20.4, *SD* = 3.1 years). Temporal principal components analyses were used to decompose ERP data in each task. Statistical analyses compared component amplitudes between stimulus type (Go vs. NoGo) and probability (High vs. Low). Neuronal source localisation was also conducted for each component. Processing Negativity was not evident; however, P1, N1a, N1b, and N1c were identified in each task, with Go P2 and NoGo N2b. The absence of Processing Negativity in this study indicated that young adults do not proactively process targets to complete the Go/NoGo task and/or questioned Processing Negativity’s conceptualisation. Additional analyses revealed stimulus-specific processing as early as P1, and outlined a complex network of active neuronal sources underlying each component, providing useful insight into Go and NoGo information processing in young adults.

## Introduction

The Go/NoGo task requires participants to respond quickly and accurately to Go (target) stimuli, while making no response to NoGo (nontarget) stimuli. Like other two-choice tasks (e.g., oddball tasks), this involves complex sensory, perceptual, and cognitive processing to discriminate between stimuli, and to regulate or control behaviour. However, Go/NoGo tasks are unique in that they provide a response set specifically for motor inhibition, the ability to suppress active or prepotent motor responses^1,2^. The purpose of this study was to clarify the early information and control processing in auditory Go/NoGo tasks by analysing the series of electroencephalographic (EEG) event-related potential (ERP) components related to the first 250 ms of Go/NoGo processing.

The first 250 ms of auditory Go/NoGo processing is generally associated with four ERP components: P1, N1, P2, and N2^3,4^. P1 is a small frontal scalp positivity that peaks ~50 ms after the onset of auditory stimuli, reflecting neuronal activity primarily generated in the temporal lobe and prefrontal cortex (Brodmann’s Area [BA] 2, 6, 22, and 24)^5,6^. P1 (or P50) is generally associated with sensory gating, an early selection mechanism involving the automatic filtering of sensory stimuli to facilitate relevant or targeted information processing^7–10^.

N1 is a large frontocentral negativity that peaks ~100 ms poststimulus, involving a complex of sensory components, including a small and diffuse N1a that peaks ~75 ms poststimulus, a frontocentral N1b at ~100 ms, and a temporal N1c at ~150 ms after stimulus onset^11–17^. These N1 components are also referred to as N1-3, N1-1, and N1-2, respectively, representing the “true” N1 components in Näätänen and Picton’s^18^ review of the N1. N1a and N1c are also considered part of the T-complex (or T-wave), a double-peaked N1 waveform that is evident at the temporal scalp electrode sites^15^.

N1 generators are located mostly in the superior temporal plane, including the primary and secondary auditory cortices (BA 41 and 42) and auditory association area (BA 22)^15,18–21^. However, N1 may also have sources in the frontal lobe (BA 9, 10, 24, 32, and 33)^22–24^, supporting links between N1 and attention^25,26^, or response selection^16,27,28^. N1 is generally considered to mark stimulus detection, and perhaps later stages of sensory gating in conjunction with P2^10,29^.

P2 is a central positivity that peaks ~200 ms poststimulus, reflecting neuronal activity in the vicinity of Heschl’s gyrus, slightly anterior to the N1 generators^30–32^. Alternate sources have also been suggested for P2, including the reticular activating system and BA 22^33,34^.

The functional significance of P2 is not clear, although suggestions have been made that it is linked to higher-level perceptual processes involved in target identification^34^. This corresponds with previous auditory ERP research illustrating differential Go and NoGo processing after N1, marked by the Go-specific P2 and NoGo-specific N2b^35^. N2b is a frontal negativity that peaks ~200 ms after NoGo stimulus onset, reflecting neuronal activity in the anterior cingulate cortex (BA 32 and 33) commonly associated with cognitive control^4,36,37^.

In auditory discrimination tasks, the automatic sensory components may be overlapped by Processing Negativity (PN), an endogenous slow wave associated with selective attention^38^. PN is considered to index a matching process between attended sensory input and an actively-maintained neuronal representation or trace of relevant target information^25,38,39^. Maintaining a trace is effortful, although it is thought to facilitate the processing of the relevant stimulus input^38^. In view of that, PN may be considered as a putative marker of proactive information processing, which could provide useful insight into the cognitive strategy that individuals are using in a task.

PN is traditionally quantified in oddball tasks as a frontocentral negative difference (Nd) between target and nontarget ERP data, and may involve an early and late component^40,41^. The early auditory PN occurs between 50–250 ms and is hemispheric in its distribution when quantified with temporal principal components analysis (PCA)^42^, consistent with suggestions that the early PN is generated in sensory-specific areas^18,43,44^; note, however, that a more recent examination of Nd indicated that the early PN is generated in the frontal lobe^22^.

Previous ERP/PCA research has identified an early hemispheric PN in auditory equiprobable Go/NoGo tasks at ~160 ms poststimulus, suggesting that participants proactively select or identify target information in that paradigm^3,35,45^. However, recent research comparing auditory oddball and equiprobable Go/NoGo processing has questioned the identity of that component^46^.

According to Attentional Trace Theory, PN should increase with target probability, representing sensory reinforcement of the attentional trace, as shown using Nd^47^. In contrast, Fogarty *et al*.^46^ found that the early hemispheric PN increased as stimulus probability decreased. However, it was suggested that the hemispheric negativity identified in that task may not represent the traditional PN, but rather N1c, which had not been identified in the auditory Go/NoGo paradigm. Accordingly, the presence of PN in that task is also unclear; this has important implications for auditory Go/NoGo processing, as the absence of the PN could indicate that young adults are not proactively processing target stimuli in that task.

The purpose of this study was to clarify the early information and control processing associated with auditory Go/NoGo tasks. To do so, this study first aimed to identify the traditional PN (Nd) in healthy young adults who completed both an ‘equiprobable’ and ‘frequent Go’ variant of the auditory Go/NoGo task. The difference between these tasks was in the probability of Go stimuli, which was expected to facilitate the characterisation of the hemispheric negativity and the identification of PN.

To gain further insight into early Go/NoGo processing, this study also aimed to explore the active neuronal sources, and stimulus type and probability effects associated with the series of temporal PCA-derived ERP components in the first 250 ms of task processing; that is, P1, N1, P2, and N2b. This was expected to provide a more detailed account of the sequential processing of auditory information in the Go/NoGo task, and of the discrete ERP/PCA components that are commonly used to study information and control processing in two-choice tasks.

Healthy young adults were expected to show a traditional PN, marked by an Nd in the 50–250 ms poststimulus period in the Go/NoGo ERP difference waveforms, indicating that young adults were proactively processing target information. Nd was hypothesised to increase with Go probability, consistent with Alho *et al*.^47^ and the theories relating PN to selective attention^25,38,39^. The PCA-derived hemispheric negativity identified in Fogarty *et al*.^46^ was hypothesised to match N1c, a temporal negativity that is maximal over the right hemisphere, corresponding to the second negative peak in the T-complex^15^. N1c amplitudes have been shown to decrease in predictable conditions^17^; thus, the hemispheric negativity was also expected to decrease as stimulus probability increased, supporting its identification as N1c, and its distinction from PN. No additional hypotheses were made regarding the other components (or analyses) included in this study.

## Results

### Trial and behavioural outcomes

There was no significant difference between the mean percentage of Go trials accepted in the equiprobable (*M* = 93.2, *SD* = 4.3%) and frequent Go conditions (*M* = 93.3, *SD* = 3.3%) after error and artefact rejection; *t*[59] = −0.08, *p* = 0.936. On average, a larger proportion of NoGo trials were accepted in the equiprobable (*M* = 95.0, *SD* = 4.2%) compared to the rare NoGo condition (*M* = 90.5, *SD* = 7.2%); *t*[59] = 6.36, *p* < 0.001. The behavioural performance outcomes are summarised in Table 1. Mean Go RTs were significantly shorter in the frequent Go condition; *t*(59) = 4.32, *p* *< *0.001. The G70/N30 task was also associated with higher rates of NoGo commission errors (*t*[59] = −7.65, *p* < 0.001), and Fast RT errors (*t*[59] = −1.82, *p* = 0.036).

**Table 1:** GM (and SD) for the behavioural outcomes by task.

	Error Rates (%)				Go Response Time (ms)	
	Commissions**	Omissions	Fast RTs*	Slow RTs	Mean**	ISD
G50/N50	3.46 (2.71)	1.62 (2.98)	0.23 (0.42)	3.98 (1.06)	364.49 (50.50)	75.87 (24.18)
G70/N30	8.62 (6.92)	1.31 (2.07)	0.38 (0.47)	3.94 (1.10)	345.00 (58.53)	74.24 (30.81)

NB. ISD = intra-individual standard deviation; *significant at p < 0.05; **significant at p < 0.001

### Raw ERP outcomes

**Figure 1:**
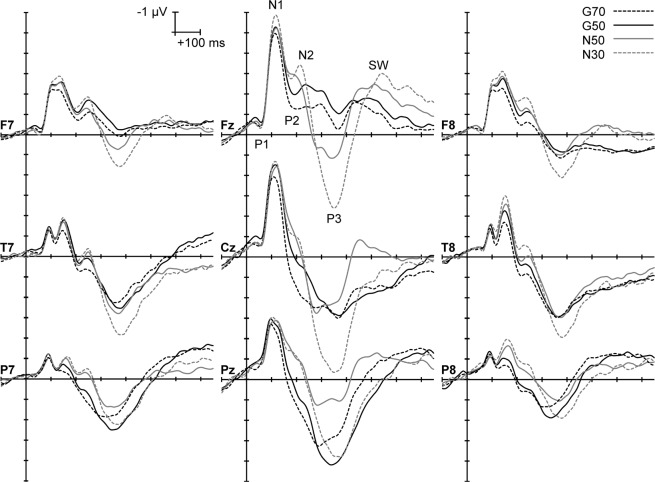
GM Go/NoGo ERPs in each condition at nine distinctive scalp sites; scalp locations are labelled in bolded text adjacent to each plot, and major ERP components are marked at Fz.

Figure 1 depicts the GM raw ERPs in each condition. At each level of stimulus probability, Go/NoGo stimulus onset is followed by a minor positive-going P1 wave that peaks ~60 ms poststimulus. P1 is followed by a major N1, involving a dominant frontocentral N1b at ~120 ms, and a T-complex represented by the negative “double-peak” between 80 and 160 ms at the temporal scalp sites (see T7 and T8 in Fig. 1); the two negative peaks in the T-complex are considered to reflect N1a and N1c, respectively. Go P2 was evident ~190 ms poststimulus, followed by N2c, P3b and a target Slow Wave (SW); whereas NoGo N2b peaked at ~220 ms poststimulus, and was succeeded by P3a, and a nontarget SW. No evidence of Nd was found in the Go/NoGo difference waves computed for each task (see Supplementary Material). Hence, the subsequent analyses focused solely on the ERP components derived using temporal PCAs.

### PCA outcomes

**Figure 2:**
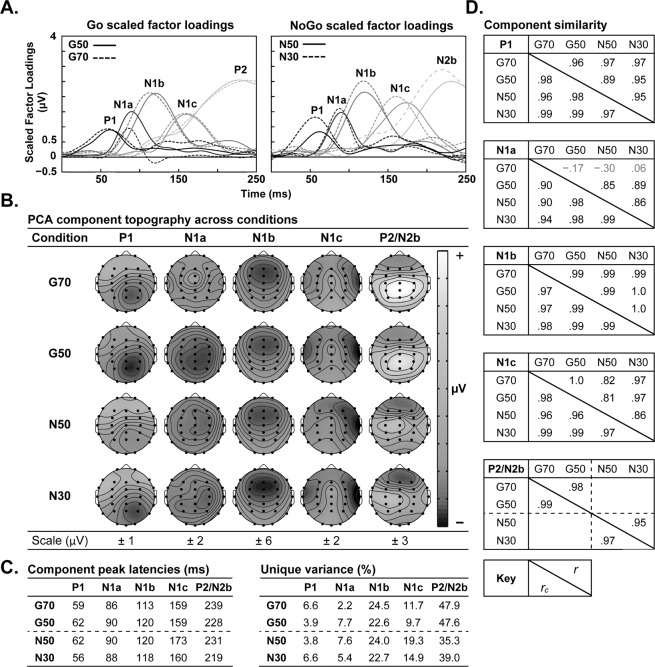
The scaled factor loadings (**A**), peak topography (**B**), peak latency and variance (**C**) for each PCA component identified in this study. The similarity of the components matched between conditions is summarised on the right (**D**), with topographical correlations (*r*) and congruence coefficients (*r*_*c*_) above and below the diagonal, respectively; correlation coefficients in grey text were not statistically significant (i.e., *p* > 0.05).

The PCA components identified in this study are depicted in Fig. 2. Five components were identified in each condition, including P1, N1a, N1b, and the hemispheric negativity, tentatively labelled N1c; P2 and N2b were also identified in the Go and NoGo conditions, respectively. Together, the five identified components accounted for ≥ 88.6% of the ERP variance within each condition. However, as indicated in Fig. 2C, three components were identified below threshold, including P1 (Factor 5) in G50 and N50, and N1a (Factor 6) in G70. The statistics in Fig. 2D, above the diagonal, show that the peak topography of each component was highly similar across conditions (*r*[28] ≥ 0.81, *p* < 0.001), excluding G70 N1a, which did not correlate with its counterparts. The congruence coefficients, below the diagonal, show that the temporal morphology of each component (including G70 N1a) was highly similar or equivalent across conditions (*r*_*c*_[248] ≥ .90, *p* < 0.001).

### Verification of the N1 components

**Figure 3:**
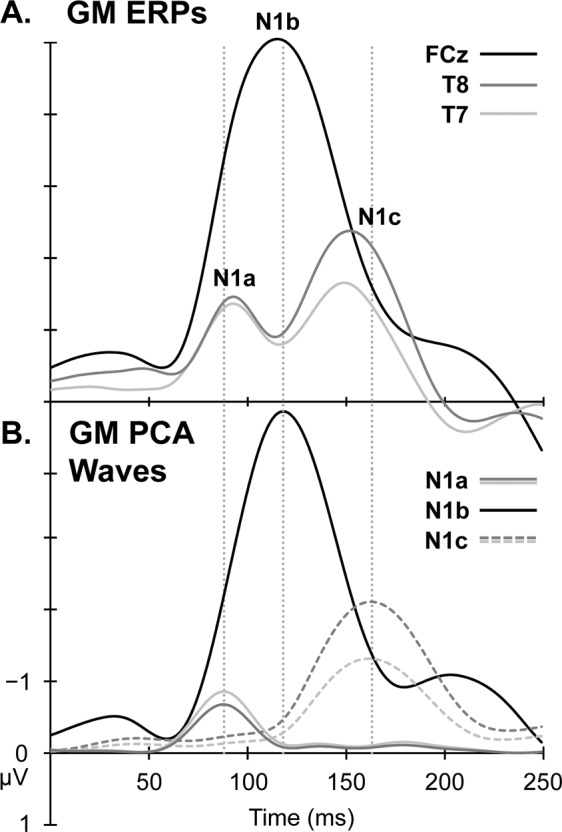
The GM raw ERPs (Part A) and PCA-derived N1 waveforms (Part B) over the 0–250 ms poststimulus period. The major N1b was represented using data at FCz (Black). The T-complex, including N1a and N1c, was distinguished at left and right temporal electrode sites; T8 (Dark Grey) and T7 (Light Grey), respectively.

Figure 3 provides a comparison of the GM raw and PCA-derived N1 components at three electrode sites distinguishing the major frontocentral N1 wave (FCz), and the T-complex (T7 and T8). As expected, the PCA-derived hemispheric negativity (i.e., N1c, represented by dashed lines in Part B) was larger over the right hemisphere, and corresponded with the second negative peak in the T-complex. The GM PCA-derived N1a and N1b also paralleled the N1a and N1b in the raw ERP data, supporting the identification of those N1 components.

### Neuronal sources

**Figure 4:**
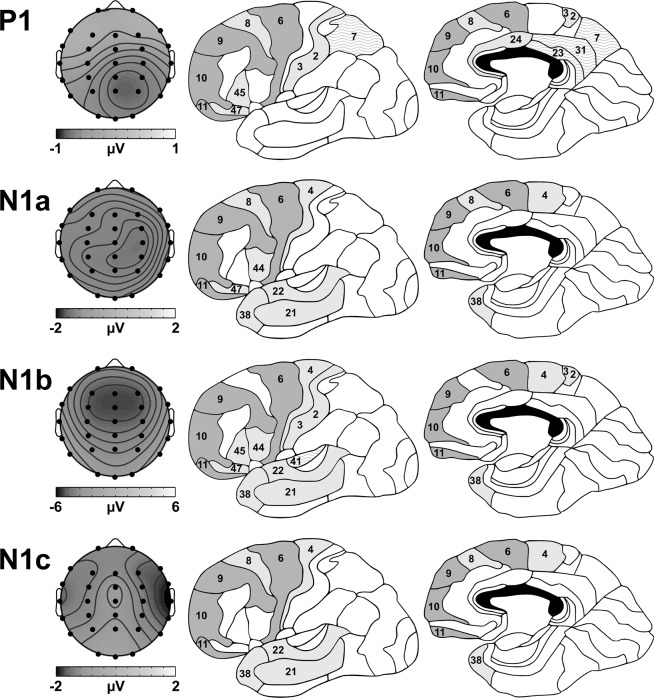
GM peak topography and Brodmann Areas (BAs) associated with the P1 and N1 components. Dark grey BAs were active in each component; light grey areas were active in multiple components; wavy areas were uniquely active in that component.

Figure 4 shows the GM peak topography and neuronal sources associated with the P1 and N1 components identified in this study. The neuronal sources of P1 were located primarily in the frontal and parietal lobes, as well as sub-lobar regions, and the temporal, occipital, and limbic lobes. In order of descending intensity, P1 sources were active in the precuneus, cingulate gyrus, inferior frontal gyrus, superior temporal gyrus, middle frontal gyrus, postcentral gyrus, medial frontal gyrus, and insula, collectively accounting for 54.3% of the voxel data variance. The most active BAs, explaining 90.8% of the P1 activation in those structures, included (in descending order) BA 7, 13 (not visible in Fig. 4), 31, 6, 10, 47, 24, 11, 9, 3, 45, 23, 8, and 2.

N1a sources were located predominantly within the frontal and temporal lobes, but were also evident in the parietal and occipital lobes. In descending order, the most active N1a sources were in the superior temporal gyrus, middle frontal gyrus, superior frontal gyrus, medial frontal gyrus, middle temporal gyrus, precentral gyrus, and inferior frontal gyrus, together explaining 54.8% of the total voxel variance. In intensity order, the BAs accounting for 90.2% of the N1a activation in those structures included BA 6, 21, 10, 38, 47, 22, 9, 11, 4, 8, and 44.

N1b sources were identified primarily in the frontal and temporal lobes, as well as sub-lobar areas, and the parietal, and occipital lobes. Beginning with the most active structures, N1b sources were located in the superior temporal gyrus, insula, inferior frontal gyrus, precentral gyrus, postcentral gyrus, middle temporal gyrus, and middle frontal gyrus, collectively accounting for 51.7% of the variance. The most active BAs, explaining 90.0% of the N1b activation in those structures, included (in descending order) BA 13, 38, 47, 21, 6, 22, 4, 3, 2, 44, 9, 11, 45, 10, and 41.

N1c sources were located predominantly within the frontal and temporal lobes, but also in the occipital and parietal lobes. The most active N1c sources (in descending order) were in the middle frontal gyrus, superior temporal gyrus, precentral gyrus, superior frontal gyrus, middle temporal gyrus, and medial frontal gyrus, explaining 51.5% of the variance in N1c voxel data. The BAs contributing to 90.8% of the N1c activation in those locations were, in intensity order, BA 6, 21, 8, 22, 10, 9, 11, 38, and 4.

**Figure 5:**
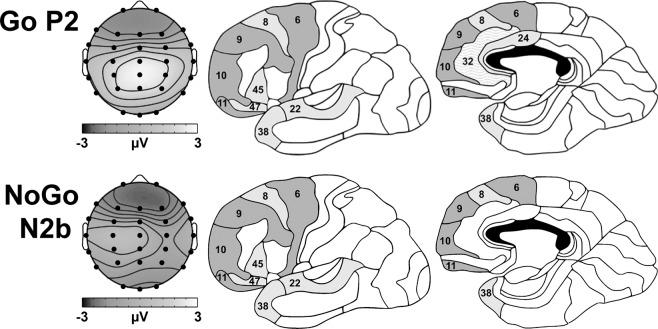
GM peak topography and Brodmann Areas (BAs) associated with the Go P2 and NoGo N2b. Dark grey BAs were active in each component; light grey areas were active in multiple components; wavy areas were uniquely active in that component.

Figure 5 illustrates the GM peak topography and neuronal sources related to Go P2 and NoGo N2b in this study. The neuronal sources of the Go P2 were primarily in the frontal, temporal, and limbic lobes, with the most active structures including (in descending order) the superior frontal gyrus, medial frontal gyrus, inferior frontal gyrus, superior temporal gyrus, middle frontal gyrus, and cingulate gyrus, together explaining 53.1% of the variance. The most active BAs accounting for 92.3% of the P2 activation in those structures were, in intensity order, BA 6, 8, 9, 47, 38, 10, 32, 24, 11, 22, and 45.

N2b sources were located mainly within the frontal and temporal lobes, with the most active structures (ordered by amplitude) including the superior frontal gyrus, inferior frontal gyrus, superior temporal gyrus, medial frontal gyrus, and middle frontal gyrus, collectively explaining 51.7% of the voxel data variance. The most active BAs accounting for 93.2% of the N2b activation in those structures included, in order of their contribution, BA 6, 47, 8, 38, 9, 10, 11, 22, and 45.

### Stimulus type and probability effects

The GM component amplitudes in each condition are summarised in Table 2. The repeated measures MANOVAs showed a main effect of stimulus type was found on P1, *F*(1,59) = 6.48, *p* = 0.014, η_p_^2^ = 0.10, with larger amplitudes following NoGo stimuli (*M* = 0.24, *SD* = 0.84 µV), relative to Go (*M* = 0.04, *SD* = 0.70 µV). N1a varied significantly with stimulus probability, *F*(1,59) = 11.80, *p* = 0.001, η_p_^2^ = 0.17, with larger N1a amplitudes associated with lower stimulus probability (*M* = −1.4, *SD* = 1.8 µV), compared to higher probability (*M* = −0.9, *SD* = 1.4 µV). That probability effect was larger for Go, than NoGo N1a amplitudes, with a significant interaction effect, *F*(1, 59) = 10.14, *p* = 0.002, η_p_^2^ = 0.15. NoGo N1b was significantly larger (*M* = −4.6, *SD* = 2.2 µV), than Go N1b (*M* = −4.2, *SD* = 2.0 µV), *F*(1, 59) = 8.33, *p* = 0.005, η_p_^2^ = 0.12; this effect was greater when stimulus probability was lower, apparent in a significant interaction, *F*(1, 59) = 8.36, *p* = 0.005, η_p_^2^ = 0.12. A main effect of stimulus probability was found on N1c, *F*(1, 59) = 15.43, *p* < 0.001, η_p_^2^ = 0.21, with larger amplitudes associated with lower stimulus probability (*M* = −1.9, *SD* = 1.1 µV), compared to higher probability (*M* = −1.5, *SD* = 1.0 µV). Go P2 amplitudes were significantly larger when Go probability was higher (*M* = 2.8, *SD* = 2.9 µV), than when Go probability was lower (*M* = 2.1, *SD* = 3.1 µV); *F*(1, 59) = 8.63, *p* = 0.005, η_p_^2^ = 0.13. No significant effects were found for the NoGo N2b.

**Table 2:** GM component amplitudes (and SD) by stimulus type and probability.

Probability	Go		NoGo	
	Higher	Lower	Higher	Lower
P1	0.15 (0.66)	−0.07 (0.72)	0.20 (0.63)	0.27 (1.00)
N1a	−0.61 (1.02)	−1.50 (1.74)	−1.22 (1.63)	−1.25 (1.83)
N1b	−4.36 (2.15)	−4.12 (1.90)	−4.26 (1.93)	−4.94 (2.35)
N1c	−1.48 (0.82)	−1.77 (0.92)	−1.58 (0.22)	−1.95 (1.22)
P2	2.83 (2.89)	2.10 (3.12)		
N2b			−1.51 (2.38)	−1.72 (2.96)

*NB*. GM component amplitudes are in µV. P2 and N2b were Go and NoGo specific, respectively.

## Discussion

This study analysed the first 250 ms of ERP data in two Go/NoGo tasks, to clarify early auditory Go/NoGo processing, and the presence of an early Go/NoGo PN in healthy young adults. No early frontal Nd was identified, and the hemispheric negativity identified in previous PCA studies matched N1c, demonstrating that there was no PN evident in young adults completing either equiprobable or frequent Go variants of the auditory Go/NoGo paradigm. Further analyses revealed complex neuronal source activations and stimulus effects throughout the Go/NoGo processing sequence, perhaps providing some direction for future models of auditory information processing.

In this study, the early PN (Nd) was expected to be evident in the Go/NoGo ERP difference waveforms between 50–250 ms poststimulus if participants were proactively processing target stimuli. No PN was identified during that period, although a frontal negativity was evident ~300 ms poststimulus, representing the difference between NoGo P3a and Go P3b (see Supplementary Material). NoGo P3a increases with decreasing NoGo probability^48^, which begs the question as to whether this P3 difference explains the traditional findings showing Nd to increase with Go probability^47^. This highlights the difficulty of interpreting ERP outcomes determined using difference waves. Despite that, the absence of Nd in this study shows that the traditional PN was not evident in young adults completing the auditory Go/NoGo task.

As hypothesised, the PCA-derived hemispheric negativity was a close representation of N1c; a temporal negativity that is larger over the right hemisphere, corresponding with the second negative peak in the T-complex^15,16^. The hemispheric negativity also decreased in amplitude as stimulus probability increased, replicating the findings in Fogarty *et al*.^46^. This also follows previous research linking smaller N1c amplitudes to more predictable stimuli^17^, providing further confirmation that the hemispheric negativity represents N1c, rather than PN. Together, with the absence of Nd, this suggests that young adults were not proactively (or selectively) processing target stimuli in either Go/NoGo variant.

This study replicated the ERP components associated with early auditory processing in a range of cognitive tasks (i.e., P1, N1, P2, and N2). Using PCA to decompose the early sensory period also enabled the clear separation of the true N1 components; including N1a, N1b, and N1c^11–16,18^. Accordingly, successful auditory processing in this task was linked to a frontal P1, a small centroparietal N1a, large frontocentral N1b, and a temporal N1c. Distinctive Go and NoGo processing was evident after N1c, marked by the subsequent Go P2 and NoGo N2b^35^.

A range of neuronal sources were linked with the Go/NoGo processing series in this study, including several frontal sources that were common to P1, N1a, N1b, N1c, Go P2 and NoGo N2b (i.e., BAs 6, 9, 10, and 11). This may be consistent with a parallel distributed processing framework^49^, and suggests that Go/NoGo processing involves a core frontal network that is active throughout the first 250 ms, together with additional sources specific to each component/processing stage. That core network may represent the cognitive control functions required throughout the task, perhaps including the coordination and integration of discrete cognitive operations, the maintenance of task goals in working memory, and behavioural regulation^50–53^.

P1 was related to activity in frontal and parietal lobes, as well as sub-lobar regions, and temporal, occipital, and limbic lobes; corroborating (and extending) previous findings linking P1 to activation in frontal and temporal areas of the brain^5,6^. The parietal and sub-lobar activation in BAs 7, 23, and 31 were unique to P1, perhaps signifying an early shift in attentional focus^54^. Together with the involvement of the core frontal network, these outcomes support the link between P1 and auditory sensory gating^7–10^. P1 was also larger to NoGo, illustrating early stimulus-specific processing, perhaps consistent with that interpretation; however, this finding should be viewed with caution due to the small mean P1 peak amplitudes, particularly in G50 (see Table 2).

N1a activity was localised mainly in the frontal and temporal lobes, but also in some parietal and occipital areas^18^. Unlike P1, no BAs were unique to N1a, relative to the other components. However, notably the frontal BAs 8 and 47 were active in relation to N1a and the preceding P1, reflecting continued processing in areas related to working memory^55,56^, and behavioural control^57,58^. N1a also represented the initial activation of several regions that were common to later processing stages (i.e., BAs 4, 21, 22, 38, and 44); these BAs have been related to a range of functions, including (but certainly not limited to) auditory processing^59^, and motor control^60,61^.

N1b was associated with activation in several structures common to P1 (BAs 2, 3, 13, and 45), and the immediately preceding N1a (BAs 4, 21, 22, 38, 44, and 47), representing the continuation of stimulus (and likely, response) processing in those areas. N1b was uniquely related to activation in BA 41, consistent with its connection to basic auditory processing, and the more general observation that N1 is generated within the primary auditory cortex^18^. It is remarkable that the primary auditory cortex was not active earlier (or later) in the auditory Go/NoGo processing sequence; perhaps this suggests that auditory N1b is the primary marker of tone frequency discrimination^62^, or the processing of stimulus offset^25,63^.

N1c was linked to activation in frontal and temporal areas common to both P1 and N1a (BA 8), and the previous N1b (BAs 4, 21, 22, and 38). This is consistent with suggestions that N1a, N1b, and N1c reflect processing in similar cortical areas^18^; indeed, BAs 4, 21, 22, and 38 were common to all three N1 components. More notably, however, is that of those cortical areas, activations in the primary motor cortex (BA 4) and the middle temporal gyrus (BA 21) were exclusive to the N1 components in this study. Together, with the frontal N1 source activations confirmed in this study, these outcomes support earlier research that proposed links between N1 and response processing in choice/RT tasks^16,27,28^.

Both N1a and N1c were larger when stimuli were rare; whereas, N1b was larger following NoGo stimuli, similar to P1. The common N1 sources and the interaction effects noted in the results could signify some functional overlap or crosstalk between these components, however, the main effects identified here could help distinguish the functional specificity of N1b and the T-complex; comprising N1a and N1c. Namely, that N1b is sensitive to stimulus type (or significance), while the T-complex is related to stimulus probability (or predictability)^17,39^.

Go P2 and NoGo N2b were both active in BAs 8, 22, 38, 45, and 47, implying some continued information processing in the frontal and temporal areas associated with P1 and N1. Additionally, P2 was also active in BA 24, and uniquely, BA 32; representing the ventral and dorsal anterior cingulate, respectively. P2 was also larger when Go probability was higher (as in Fogarty *et al*.^46^). Together, these outcomes corroborate the suggestion that auditory P2 is (at least) partly generated in the temporal lobe^33,34^. Its link to the anterior cingulate could also substantiate its relationship with sensory gating or attention^10,64^, which was perhaps enhanced by increasing the predictability of Go stimuli.

This study suggests that the temporal PN (or N1) identified in previous PCA studies was N1c. From that viewpoint, those earlier studies indicate that larger N1c amplitudes are associated with caffeine consumption^65,66^, shorter oddball RTs^67^, and the processing of tonal stimuli (vs. phonetic stimuli)^68^. Previous studies would also suggest that N1c is more enhanced at temporal sites (relative to the midline) following Go stimuli, although that may be because the NoGo counterpart was often more negative at frontal-midline sites^3,35,45,65,69,70^. These observations, and the present findings, strongly support a link between N1c and stimulus-response processing, at least in paradigms that require a response. Moreover, the clarification of those effects could provide useful insight for researchers using the T-complex to study auditory perception or deficits in individuals with learning difficulties (e.g., dyslexia)^71–73^.

The absence of PN in this study was considered to show that young adults were not proactively processing target stimuli, following theories suggesting that PN represents activity associated with an attentional trace^38^, stimulus set^74^, or prediction of target stimulus input^39^. However, that does not discount the possibility of proactive *response* processing. Indeed, Go primacy effects were identified in this study, as signified by the shorter RTs and higher commission error rates in the frequent Go (vs. equiprobable) variant of the Go/NoGo task. Hence, the present findings tentatively suggest that increasing stimulus probability can prime response processes separately from sensory processing. Alternatively, the present findings could question the traditional view of PN as a marker of early, proactive, or selective information processing.

Several limitations in this study can be addressed in future research. Firstly, this study was limited to the first 250 ms of task processing, which aided the PCA extraction of the early ERP components that were the focus of this study; however, it would be useful to apply the same analyses to later time periods so that the present findings can be considered relative to the broader task processing sequence. Source analyses should also be conducted on the Go and NoGo P1 and N1 components separately. In this study, source analyses were conducted on GM components, preventing the detection of possible Go/NoGo source differences that might help to elucidate the early stimulus-specific effects on component amplitudes. Including a classic oddball task would also have been useful to verify the traditional PN (Nd) in the current sample, and to strengthen the conclusions in this study by providing a PN for comparative purposes.

The ERP source outcomes in this study also indicate that each component represents complex neuronal activations that could be consistent with a parallel distributed processing framework, which posits that information processing occurs as activity propagates through a system of connected modules (i.e., neuronal sources)^49^. Accordingly, analysing the functional connectivity between the active areas identified in each component could potentially further our understanding of the discrete processing stages in auditory Go/NoGo tasks. That approach could also assist in the confirmation of the core network of (pre)frontal areas identified in this study, and assist in clarifying its role (and that of other brain areas) in the sequential processing of auditory information.

This study clarified the early ERP/PCA component series associated with auditory Go/NoGo sensory processing in young adults. As expected, the hemispheric negativity identified in previous ERP/PCA research was a marker of N1c. Together with the absence of the traditional PN (Nd), this suggests that young adults did not proactively process the target stimulus input in this paradigm. However, the behavioural outcomes showed that the Go response was still primed by increasing target probability; this has interesting implications for the cognitive control of both stimulus and response processing. A complex of neuronal generators was associated with each component/processing stage identified in this paradigm. In future, these observations could provide a useful basis for models of auditory information and control processing in healthy young adults.

## Methods

### Participant demographics and screening

Sixty healthy young adult university students volunteered for this study in return for course credit (31 female; *M* = 20.4, *SD* = 3.1 years), through the University of Wollongong School of Psychology Research Participation Scheme. Before testing, each participant gave their informed consent and was assessed against key exclusion criteria: those with ongoing mental health issues, pre-existing central neurological complaints, or head injuries causing unconsciousness, were excluded, along with those who had consumed psychoactive substances (≤12 hours), or caffeine/tobacco (≤4 hours) before testing. Participants were also required to be right-handed, which was assessed using the Edinburgh Handedness Inventory^75^. This research was completed in accordance with a protocol approved by the University of Wollongong and Illawarra Shoalhaven Local Health District Human Research Ethics Committee.

### Physiological recording

Continuous electrophysiological data, from DC to 30 Hz, were recorded throughout each task using a Neuroscan Synamps2 amplifier (sampling rate: 1000 Hz). EEG data were recorded from 30 scalp sites (Fp1, Fp2, F7, F3, Fz, F4, F8, FT7, FC3, FCz, FC4, FT8, T7, C3, Cz, C4, T8, TP7, CP3, CPz, CP4, TP8, P7, P3, Pz, P4, P8, O1, Oz, O2) and the right mastoid, grounded at AFz and referenced to the left mastoid. EOG data were also recorded with four electrodes placed beside the outer canthi, and above and below the left eye. Non-polarisable sintered Ag/AgCl electrodes were used for cap and EOG electrodes, with impedances below 5 kΩ.

### Task and procedure

Participants were first seated in a darkened sound-attenuated room to complete a brief EOG calibration task^76^. Afterwards, participants received equipment and instructions for two auditory Go/NoGo tasks, each involving two blocks of 150 uncued Go/NoGo tones (1000 or 1500 Hz). Tones were presented through circumaural headphones at 60 dB SPL (calibrated by an artificial ear and sound level meter: Brüel & Kjær, model 4152), using a stimulus-onset asynchrony (SOA) of 1250 ms. The duration of each tone was 80 ms, including 15 ms rise/fall times. The tone (i.e., trial) order was shuffled prior to each block, and the Go and NoGo tone frequencies were counterbalanced across blocks, within each task. The only difference between these two tasks was the global stimulus probability: in one task, Go and NoGo tones were *equiprobable* (*p*[Go] = 0.5); in the other, Go tones were more *frequent* (*p*[Go] = 0.7). Task and block order were counterbalanced across participants.

Participants were instructed to respond to the Go tone as quickly and accurately as possible, whilst ignoring the other (NoGo) tone. All responses had to be made with a button-press with the right thumb, using a Logitech® Precision Gamepad Controller. An example of the Go tone, and a short practice, was provided before each block. Ten random trials were presented in each practice, with the same Go tone and stimulus probability as the subsequent block; practice blocks were repeated if necessary.

### Measure quantification

#### Behavioural performance

Individual mean response time (RT) was calculated across Go trials in each task. RTs exceeding 2 *SD* above or below the mean RT were classified as Slow or Fast RT errors, reflecting unusually delayed or impulsive responses, respectively. Mean RT and intra-individual standard deviation of RT (ISD) were recalculated after erroneous or artefactual trials were rejected (see the next Methods section, *ERPs*), to ensure that these measures reflected only correct/accepted Go trials. Go omission and NoGo commission error rates were also recorded to assess Go and NoGo accuracy.

#### ERPs

After EOG-correcting the raw EEG data using the regression approach established by Croft and Barry^76^, the data were re-referenced to digitally linked mastoids, and lowpass filtered to 25 Hz (FIR, 24 dB/Octave, zero phase shift) in Neuroscan (Compumedics, v. 4.5). Go and NoGo trials were first separated into full epochs ranging from −100 to +750 ms relative to stimulus onset, and then baselined using their prestimulus period. Any epochs containing incorrect responses, or artefact exceeding ±100 µV at any electrode, were rejected. The remaining trials were then averaged across blocks to form Go and NoGo ERPs for each participant in each task, resulting in four ERP datasets separated by stimulus type (i.e., Go vs. NoGo) and stimulus probability (i.e., Higher vs. Lower): equiprobable Go (G50), equiprobable NoGo (N50), frequent Go (G70), and rare NoGo (N30). Difference waveforms were then computed within subjects by subtracting the averaged NoGo ERP data from the mean Go data within each task; these waveforms were then examined for Nd.

Following Barry *et al*.^77^, separate temporal PCAs were conducted on a restricted 0–250 ms period of each ERP dataset in Matlab (The Mathworks, v. 8.0, R2012b), to enhance the extraction of the early auditory ERP components. This process was implemented using the erpPCA functions provided by Kayser and Tenke^78^ (http://bit.ly/2oX0etA), adjusted to omit the subtraction of the grand mean (GM) ERP^79^. Each PCA was implemented using the covariance matrix with Kaiser normalisation, and unrestricted Varimax rotation, and included 1800 cases (60 participants × 30 sites) and 250 variables (timepoints). PCA factors explaining ≥5% of the ERP variance were output in variance order (largest to smallest), and were manually identified as ERP components according to their topography and latency; this process was guided by the preceding ERP literature (as outlined in the Introduction). If an expected component (i.e., P1, N1, P2, or N2) was not extracted in a condition at first, it was searched for below the variance cut-off (down to ≥2%) if it met the initial threshold in another condition.

### Statistical analysis

Behavioural performance outcomes were compared between tasks using paired sample *t*-tests. Following Barry *et al*.^77^, matching components were compared to determine whether the same (or similar) components were extracted within each dataset. Tucker’s^80^ congruence coefficients (*r*_*c*_) were calculated between the unscaled factor loadings of matching components to assess their temporal similarity; components are considered temporally equivalent if *r*_*c*_ ≥ 0.95, and highly similar when 0.85 ≤ *r*_*c*_ ≤ 0.94^81^. Simple correlations were also calculated between component amplitudes (at each of the 30 sites) to assess their topographic similarity. GM components were then formed for further analyses by averaging matching PCA component waveforms.

### Stimulus type and probability

Two-way repeated measures MANOVAs were used to analyse stimulus type (Go vs. NoGo) and stimulus probability (Higher vs. Lower) effects on the peak component amplitudes in each dataset. Individual peak component amplitudes were computed within each dataset as an average across the electrodes marking the component’s key topographical features, based on the peak electrode sites and contour lines in the GM component headmaps. This approach helped to minimise the influence of any random error that could be attributed to a single site^82^. Each *F*-test had (1, 59) degrees of freedom with statistical significance determined at α < 0.05.

### Source analyses

Following the methods in Barry *et al*.^83^, the “exact” version of low-resolution electromagnetic tomography (eLORETA)^84,85^ was used to estimate the cortical sources of the GM PCA component waveforms. This process was conducted in LORETA–KEY (v. 20170220) using default settings, with no regularisation, and a threshold of 0.0000001; and exported positive and negative data. This program separates the brain into 6,239 voxels of 5 mm^3^, and outputs 3-D inverse solution locations in relation to a realistic brain atlas from the Montreal Neurological Institute (MNI); solutions are reported in voxel values in µA/mm^2^. The exported voxel values were grouped according to their structural brain location and then summed to determine the most active sources that accounted for ≥50% of the total current density for each component. The BAs that accounted for ≥90% of the activation in those structures were also reported.

## Supplementary information

Supplementary Material.

## Data Availability

The datasets generated during and/or analysed during the current study are available from the corresponding author on reasonable request.
