# Supplementary material for: The First 250 ms of Auditory Processing: No Evidence of Early Processing Negativity in the Go/NoGo Task
Source: Sci Rep. 2020 Mar 4;10:4041. doi: 10.1038/s41598-020-61060-9 (PMC7055275; doi:10.1038/s41598-020-61060-9)
Supplement: Supplementary file 1 — Supplementary Material. [file 41598_2020_61060_MOESM1_ESM.pdf]

The first 250 ms of Auditory Processing: No Evidence of Early Processing Negativity in the Go/NoGo Task

Supplementary Material

Jack S. Fogarty[1], Robert J. Barry[1], & Genevieve Z. Steiner[1, 2]

[1]Brain & Behaviour Research Institute, and School of Psychology, University of Wollongong, Northfields Avenue, Wollongong NSW 2522, Australia

[2]NICM Health Research Institute and Translational Health Research Institute (THRI), Western Sydney University, Narellan Road, Campbelltown NSW 2560, Australia

Overview

In this study, Go/NoGo ERP difference waveforms were calculated to identify the negative difference (Nd) traditionally used to quantify Processing Negativity (PN)[1,2]. Particular interest was also given to the early PN (Nd), as it was considered to reflect a matching process, indicating that participants were proactively maintaining an attentional trace (or representation) of sensory information to facilitate target processing[1,3,4].

Two auditory Go/NoGo tasks were used in this study, which required participants to respond to Go (target) tones, and ignore NoGo (nontarget) tones; these tasks were similar to those used to study PN in the past. Early PN (Nd) is a frontal negativity that occurs between 50–250 ms poststimulus; although, its latency can vary for several reasons (see Näätänen's[1] review). Accordingly, we examined the entire stimulus-locked epoch (-100 to +750 ms) for signs of Nd. Due to the task-relevance of the Go tone, PN (Nd) was expected to be identified in relation to Go stimuli; hence, difference waveforms were calculated for each subject by subtracting their mean NoGo ERP waveforms from their averaged Go ERP data. Further confirmation of PN (Nd) was expected to be shown using a stimulus probability effect, replicating the positive relationship between Nd amplitude and target stimulus probability, found by Alho et al.[2].

ERP outcomes

The Grand Mean (GM) raw ERPs and difference waveforms computed for each condition and task are displayed in Fig. S1, respectively. It is clear in the Go - NoGo difference waveforms that the 50–250 ms poststimulus period was positive across the scalp. Indeed, the GM raw frontal amplitude (averaged over F3, Fz, F4, and FCz) over that timeframe was .02 (*SD* = .54 μV) and .99 (*SD* = .69 μV) in the equiprobable and frequent-Go tasks, respectively. This finding demonstrated that there was no Nd evident in the expected time window in either task.

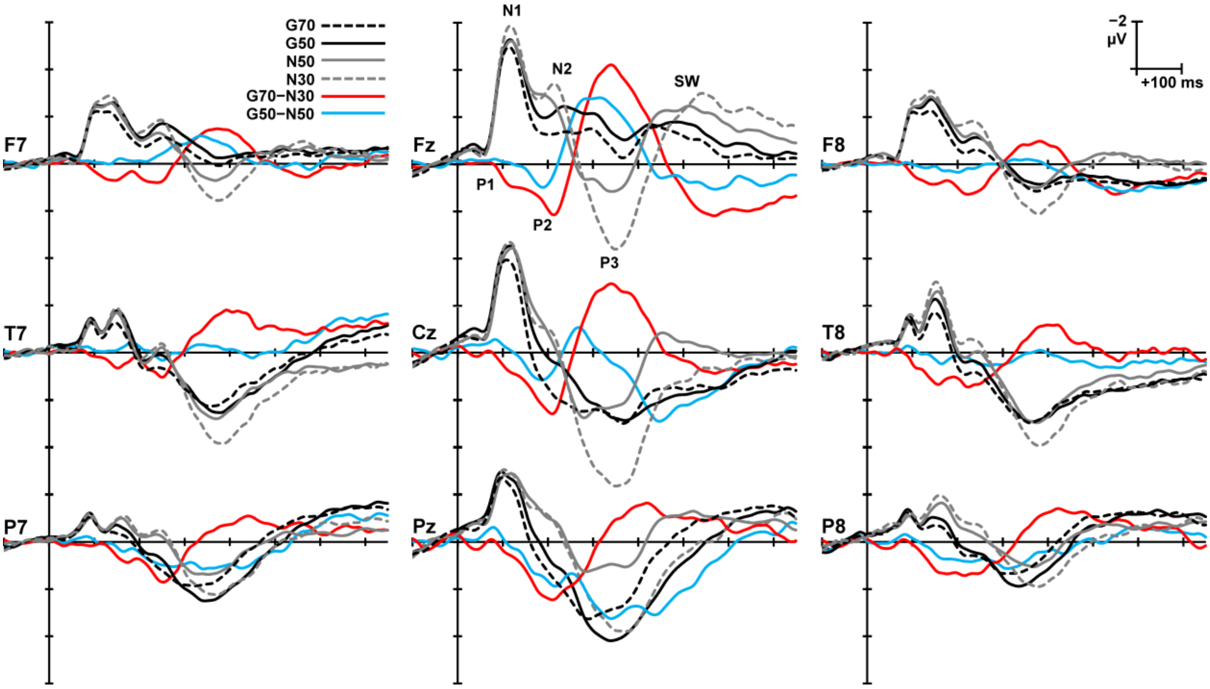

Figure S1. Grand Mean (GM) raw ERPs for each condition, and the difference waveforms calculated for the equiprobable (i.e., G50 − N50; Blue) and frequent-Go (i.e., G70 − N30; Red) variants of the auditory Go/NoGo task.

In Fig. S1 a large negative difference wave is also evident at Fz and Cz between ~ 250-450 ms poststimulus. That negative difference was tentatively considered a “late” candidate for PN (Nd); however, after closer inspection it was instead decided to reflect a relative difference between the Go and NoGo P3, given its alignment with the frontocentral NoGo P3 in each task; this is discussed briefly in the main article. Overall, these findings indicate that no traditional PN (Nd) wave was evident to target stimuli in either task.

References

1. Näätänen, R. Processing negativity: An evoked-potential reflection of selective attention. *Psychol Bull.* **92**, 605–640 (1982).
2. Alho, K., Lavikainen, J., Reinikainen, K., Sams, M. & Näätänen, R. Event-related brain potentials in selective listening to frequent and rare stimuli. *Pyschophysiology.* **27**, 73–86 (1990).
3. Hillyard, S.A. & Kutas, M. Electrophysiology of cognitive processing. *Annu Rev Psychol.* **34**, 33–61 (1983).
4. Schröger, E., Marzecová, A. & SanMiguel, I. Attention and prediction in human audition: a lesson from cognitive psychophysiology. *Eur J Neurosci.* **41**, 641–664 (2015).
